# A case of elbow hyperextension leading to complete brachial artery rupture

**DOI:** 10.1186/1749-7922-2-6

**Published:** 2007-03-01

**Authors:** Deva S Jeyaretna, Michael Butler, Huw G David, Alasdair J Walker

**Affiliations:** 1 Department of Surgery, Derriford Hospital, Plymouth. PL6 8DH, UK; 2 Department of Trauma and Orthopaedics, Derriford Hospital, Plymouth. PL6 8DH, UK

## Abstract

**Background:**

To our knowledge there are no cases in the literature of traumatic vascular injury of the brachial artery by elbow hyperextension without elbow dislocation based on either clinical or radiological evidence.

**Case presentation:**

We present the first case of complete brachial artery rupture resulting from a hyperextension injury to an elbow, without dislocation. The history, early assessment and operative treatment with figures are presented.

**Conclusion:**

We advocate prompt clinical assessment by orthopaedic and vascular teams and early surgical exploration and repair.

## Background

Brachial artery rupture is a recognised complication of closed and open traumatic elbow dislocation and there are many case reports and short series in the literature[1]. We present a case of elbow hyperextension leading to traumatic brachial artery rupture and believe this to be the first case of a brachial artery rupture following a hyperextension injury but without fracture or dislocation.

## Case presentation

A 34 year old right-handed, semi-professional rugby player suffered a hyperextension injury to his right elbow after attempting a tackle with his right arm extended. At the scene he was immediately attended by an experienced doctor who diagnosed an acute arterial injury in a rapidly swelling elbow. He was sent to the Accident and Emergency Department where he was assessed by both Orthopaedic and Vascular Surgeons within forty minutes of the injury. On examination in the Accident and Emergency Department, his forearm was pale from mid-forearm distally, the elbow was bruised and extremely swollen but the skin was intact. The distal arm and hand were cold, sensation was absent and radial and ulnar pulses were not palpable. Doppler was also unable to demonstrate presence of arterial flow. Swelling prevented palpation of the brachial pulse. There was a complete loss of motor function in the hand. Clinically, the elbow was in joint and radiographs were entirely normal. An acute brachial artery injury was diagnosed and he was taken immediately to theatre.

**Figure 1:**
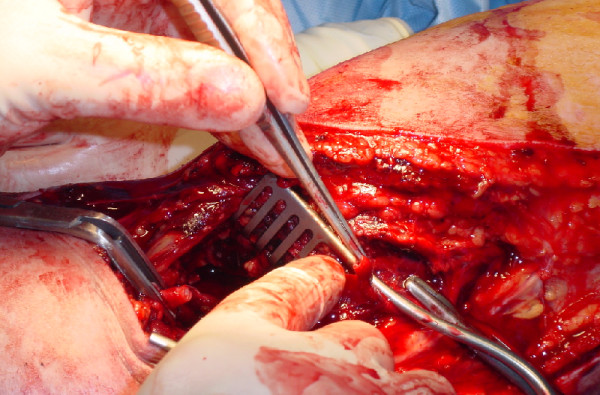
Complete transection of the brachial artery just above the level of the elbow.

**Figure 2:**
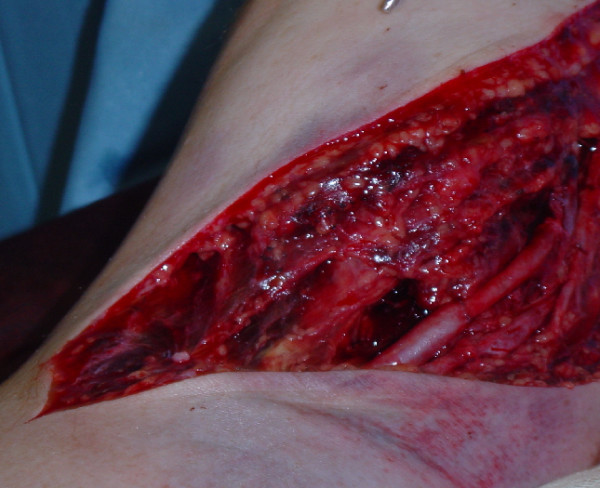
The brachial artery repaired with a segment of ipsilateral cephalic vein used as a reversed interposition graft.

Under a general anaesthetic a lazy 'S' incision was made across the ante-cubital fossa. The median nerve was bruised but intact. Biceps brachialis was partially ruptured and there was a complete transection of the brachial artery just above the level of the elbow (Figure 1). A segment of ipsilateral cephalic vein was harvested and used as a reversed interposition graft to repair the brachial artery (Figure 2). Following this repair, the hand immediately regained normal colour and warmth. Doppler examination demonstrated biphasic pulses in both radial and ulnar arteries. Primary closure of the wound was not attempted to facilitate postoperative swelling and fasciotomies were performed. A formal examination under anaesthesia demonstrated that the elbow was stable in both flexion and extension and with varus and valgus stresses. Screening radiographs with an image intensifier were also normal.

Post operatively, sensation and movement in the forearm and hand improved although there was complete loss of median nerve function. The patient was discharged home on the eighth post-operative day. Some improvement in median nerve function was noted at this stage.

## Discussion

No evidence exists in the literature of complete brachial artery injury without either open or closed elbow dislocation. There has been one case report of an occult dislocation with brachial artery disruption[2]. The force required to dislocate the elbow joint is significant and we believe that this is the first reported case of a hyperextension injury to the elbow in an extremely fit and strong semi-professional sportsman in association with a complete brachial artery rupture.

We think it improbable that this patient suffered an acute dislocation based on the examination findings and the absence of abnormal radiology. This leads us to conclude that the injury resulted purely from the momentary hyperextension of the elbow joint as described by the patient. The importance of this case lies in the development of a serious and limb-threatening closed injury in the absence of associated orthopaedic injury. We have found that in the athlete, hyperextension injury to the elbow held in extension can lead to traumatic brachial artery rupture. We would commend the value of prompt clinical assessment by orthopaedic and vascular surgical teams and advocate immediate surgical exploration and repair.

## Competing interests

The author(s) declare that they have no competing interests.

## Authors' contributions

DSJ and MB drafted the original manuscript. DSJ and AJW provided the pictures. MB and HGD provided the orthopaedic opinion and DSJ and AJW provided the vascular opinion. All authors have read and approved the final manuscript.
